# Systems Thinking and Complexity Science Methods and the Policy Process in Non-communicable Disease Prevention: A Systematic Scoping Review

**DOI:** 10.34172/ijhpm.2023.6772

**Published:** 2023-02-26

**Authors:** Chloe Clifford Astbury, Kirsten M. Lee, Elizabeth McGill, Janielle Clarke, Matt Egan, Afton Halloran, Regina Malykh, Holly Rippin, Kremlin Wickramasinghe, Tarra L. Penney

**Affiliations:** ^1^Global Food System & Policy Research, School of Global Health, York University, Toronto, ON, Canada.; ^2^Department of Health Services Research and Policy, London School of Hygiene & Tropical Medicine, London, UK.; ^3^Department of Public Health, Environments and Society, London School of Hygiene & Tropical Medicine, London, UK.; ^4^World Health Organization European Office for the Prevention and Control of Noncommunicable Diseases, Moscow, Russian Federation.; ^5^Department of Nutrition, ExercDepartment of Nutrition, Exercise and Sports, University of Copenhagen, Copenhagen, Denmark.ise and Sports, University of Copenhagen, Copenhagen, Denmark

**Keywords:** Non-Communicable Disease, Policy, Systems Thinking, Complexity Science, Population-Level Prevention

## Abstract

**Background:** Given the complex determinants of non-communicable diseases (NCDs), and the dynamic policy landscape, researchers and policymakers are exploring the use of systems thinking and complexity science (STCS) in developing effective policies. The aim of this review is to systematically identify and analyse existing applications of STCS-informed methods in NCD prevention policy.

**Methods:** Systematic scoping review: We searched academic databases (Medline, Scopus, Web of Science, EMBASE) for all publications indexed by 13 October 2020, screening titles, abstracts and full texts and extracting data according to published guidelines. We summarised key data from each study, mapping applications of methods informed by STCS to policy process domains. We conducted a thematic analysis to identify advantages, limitations, barriers and facilitators to using STCS.

**Results:** 4681 papers were screened and 112 papers were included in this review. The most common policy areas were tobacco control, obesity prevention and physical activity promotion. Methods applied included system dynamics modelling, agent-based modelling and concept mapping. Advantages included supporting evidence-informed decision-making; modelling complex systems and addressing multi-sectoral problems. Limitations included the abstraction of reality by STCS methods, despite aims of encompassing greater complexity. Challenges included resource-intensiveness; lack of stakeholder trust in models; and results that were too complex to be comprehensible to stakeholders. Ensuring stakeholder ownership and presenting findings in a user-friendly way facilitated STCS use.

**Conclusion:** This review maps the proliferating applications of STCS methods in NCD prevention policy. STCS methods have the potential to generate tailored and dynamic evidence, adding robustness to evidence-informed policymaking, but must be accessible to policy stakeholders and have strong stakeholder ownership to build consensus and change stakeholder perspectives. Evaluations of whether, and under what circumstances, STCS methods lead to more effective policies compared to conventional methods are lacking, and would enable more targeted and constructive use of these methods.

## Background

 The determinants of many non-communicable diseases (NCDs) are complex and inter-related, and policies designed to tackle them are made and unfold within dynamic realities. As such, the need for a ‘system-level’ approach to NCD prevention, which encompasses complex system properties, is increasingly recognised.^1^ Systems thinking and complexity science (STCS) represents a multi-disciplinary field of established and emergent theories and methods^2^ which may be applied to NCD prevention. While a differentiation has been drawn between STCS as distinct traditions,^3^ STCS theories and methods are broadly characterised by the idea that real-world phenomena exist within complex systems composed of dynamic actors, including people, organisations and other structures, which evolve in response to each other and their contexts.^2^ These complex systems may have characteristics such as non-linear relationships, feedback loops and delays,^4-6^ making their behaviour challenging to predict using more conventional methods for generating evidence.^7^

 STCS theories and methods may lend themselves well to addressing complexity in policy-making for NCD prevention, an area characterised by a wide range of determinants, distributed responsibility for policy areas with direct and indirect impacts on NCD outcomes, and powerful non-governmental actors shaping environments that can support or undermine NCD prevention. In addition, many STCS methods are participatory in nature, facilitating stakeholder engagement and, in some cases, consensus-building,^8,9^ both important attributes in policy-making. The potential of STCS methods for NCD prevention is evidenced by existing reports emphasising the importance of a systems perspective, such as the Lancet Report on the Global Syndemic of Obesity,^10^ the Foresight report^11^ and the World Health Organization (WHO) Global Action Plan for Physical Activity,^12^ which cites healthy systems as one of its explicit aims. Initiatives such as the Office of Behavioral and Social Sciences Research at the National Institutes of Health^13^ and the Australian Prevention Partnership Centre^14^ also highlight a commitment to a systems perspective in NCD prevention research and practice.

###  What Role Can Systems Thinking and Complexity Science Play in Public Health and Health Policy?

 Methods informed by STCS have been applied in various contexts, and health researchers have explored their utility in solving seemingly intractable public health issues. These applications in public health are growing rapidly, with as many as 90% of published examples appearing in the past decade.^15^ Several reviews have documented existing approaches to applying STCS to methods and practice in public health and health policy, with most commenting on the relative paucity of studies documenting practical applications of such methods.^2,16-18^

 While examples of practical applications are limited, reviews have demonstrated the benefits of applying methods informed by STCS to health policy. A 2015 review on the application of system dynamics modelling to support health policy at any level of government reported that a key strength of the method was its ability to engage stakeholders and facilitate consensus-building.^16^ This was achieved by inviting their participation in developing a model, resulting in agreement over the optimal policy strategy to tackle a given health problem.^16^ A 2019 review of complex systems approaches to mental health commented on the potential applications of such methods to mental health policy. The review stated that they might be particularly useful in two processes: first, determining the potential impacts of ‘distal’ policies, where the policy was removed from its potential impacts in terms of time or causality; and second, assessing what conditions might be necessary for a policy to be successful.^17^

 In addition to these advantages, STCS-informed methods have the potential to add robustness to evidence-informed policy-making. Despite the emphasis on evidence-based policy in public health,^19^ the role of research evidence in policy-making remains relatively limited,^20^ with policy-makers often differing with researchers around what sort of evidence is ‘good’ and ‘useful.’^21,22^ Further, evidence generated by researchers may only be incorporated at particular points in the policy process, with many parts of the policy process being a complex series of negotiations between different perspectives and interests. Given that policy-makers already operate in a complex and dynamic space, methods informed by STCS may support them in bringing greater rigour and transparency to the policy process, potentially leading to the implementation of more evidence-informed policy.

 While many working in policy have expressed an interest in the insights yielded by STCS methods, a recent study of policy evaluators concluded that the methods were in limited use, and that the pragmatic framing of these methods should be seen as a priority to ensure their greater penetration into the policy process.^23^ Evaluators described a number of reasons why they did not use STCS-informed methods, including the perception that simple policies and evaluations could be effective despite complex contexts; the idea that complexity was implicitly drawn out in policy and evaluation processes without the need for explicit methods; and the lack of relevant capacity and skills.^23^

## Aims and Scope

 While there has been substantial discussion and theoretical development related to the application of STCS in the policy process,^24-26^ well-documented examples of how STCS approaches can be applied to this arena, particularly at the national level and in a global context, are less common. A comprehensive and systematic review of the application of STCS-informed methods in the policy process is needed, with particular attention to specific methods used to support NCD prevention.

 Further, a gap exists in determining which of these methods are useful to particular processes and practical for practitioners with different needs and levels of resource, and in making these distinctions accessible to potential users. Scholars of complex systems have previously emphasised the importance of increasing the use of methods informed by STCS in public policy processes, and the responsibility held by researchers to effectively translate their knowledge and methods to encourage their adoption in the policy process.^27,28^ A review of existing practice which documents clear examples of how these methods can be applied in this context, as well as under what conditions a certain approach might be most useful, is an important part of this process of knowledge translation.

 Therefore, the objective of this review was to systematically identify and summarise existing applications of STCS-informed methods in NCD prevention policy, documenting key methodological elements and identifying which domains of the policy process they have been applied to.

## Methods

 We conducted a systematic scoping review of peer-reviewed literature documenting the application of methods informed by STCS to the policy process in NCD prevention. This review was not registered but is based on a previously published protocol.^29^ Results are reported in accordance with the Preferred Reporting Items for Systematic Reviews and Meta-Analyses (PRISMA-2020) statement.^30^

 The systematic scoping review was conducted according to guidelines published by Arksey and O’Malley and refined by Levac and colleagues,^31-33^ which emphasise an iterative approach for exploratory research questions.^31^

###  Stage 1: Identifying the Research Questions

 Informed by our study objective, our central research questions were:

1. How have methods informed by STCS been applied in the policy process in NCD prevention? Which domains of the policy process and areas of NCD prevention policy have methods informed by STCS been applied to?
2. What practical considerations, such as advantages, limitations, barriers and facilitators, have been described in applying STCS-informed methods to NCD prevention policy?

By policy we refer to *public *policy, defined as ‘a set of interrelated decisions taken by a political actor or group of actors concerning the selection of goals and the means of achieving them within a specified situation where those decisions should, in principle, be within the power of those actors to achieve.’^34^ We understand policy as being ultimately in the hands of government, although we recognise that a number of limitations constrain the policy options available to government, including other domestic and international actors.^34,35^ For the purposes of this review, we extended the definition of government to include regional (eg, continent-wide) and global governing bodies, as well as local and national government.

 Following Howlett and Cashore’s conceptualizations of public policy, we characterised the policy process as one which moves from broader ‘goals’ to concrete ‘means’: specifically, on-the-ground policy measures designed to achieve the stated goals.^35^ We used the definition of the domains of the policy process developed by the Centers for Disease Control and Prevention (CDC) (see Table 1).^36^

**Table 1:** The Domains of the Policy Process^a^

**Domain**	**Description**
Problem identification	Clarify and frame the problem or issue in terms of the effect on population health
Policy analysis	Identify different policy options to address the problem/issue and use quantitative and qualitative methods to evaluate policy options to determine the most effective, efficient, and feasible option
Strategy and policy development	Identify the strategy for getting the policy adopted and how the policy will operate
Policy enactment	Follow internal or external procedures for getting policy enacted or passed
Policy implementation	Translate the enacted policy into action, monitor uptake, and ensure full implementation

^a^From *Overview of CDC’s policy process.*^36^

###  Stage 2: Identifying Relevant Studies

 We systematically searched electronic databases (Medline, Scopus, Web of Science, EMBASE). The search strategy was informed by the main concepts in our research question using the Population Concept Context Framework recommended by the Joanna Briggs Institute for use in scoping reviews^37,38^:

- Population: Whole population approach to NCD prevention;
- Concept: Methods and approaches informed by STCS;
- Context: Policy-making and different domains of the policy process at different levels of government, including local, national and supranational.

We conducted searches on October 13, 2020, including studies published at any previous point in time. Search terms for the Concept included specific methods informed by STCS (eg, network analysis, causal loop diagram, group model building) as well as broader terms indicating a systems or complexity approach taken to different types of research (eg, complexity, complex system, adaptive system, system lens) (see Supplementary file 1 for detailed search).

###  Stage 3: Study Selection

 We collated and screened records identified through the searches using the online platform Covidence.^39^ Studies were included where they met all of the following criteria:

1. Primary study from any country or region, available in English;
2. Self-identify as taking an approach informed by STCS;
3. Report empirical findings from a piece of research done during a specific point in the policy process (ie, problem identification, policy analysis, strategy and policy development, policy enactment, policy implementation, evaluation, stakeholder engagement and education)^36^; and
4. Focus on a subject related to NCD prevention.

Titles and abstracts were initially screened by one researcher (CCA) against these inclusion criteria. Full texts of potentially relevant records were then independently assessed against these criteria by two researchers (CCA, along with TP or EM). Discrepancies were resolved by discussion with reference to the inclusion criteria.

###  Stage 4: Charting the Data

 We conducted data charting, a process which parallels data extraction in Arksey and O’Malley’s method for scoping reviews,^31^ using a data charting form designed to identify the information required to answer the research questions (see Supplementary file 2). As recommended, we piloted the data charting form with ten records to ensure that it was consistent with the research questions.^31-33^ One researcher undertook data extraction (JC), and a second researcher checked the extracted data (CCA or KML). Discrepancies were resolved through discussion.

###  Stage 5: Collating, Summarising and Reporting the Results

 We undertook quality assessment of the included studies using a novel quality assessment tool informed by the approach developed by Dixon-Woods and colleagues as part of their critical interpretive synthesis, where studies are excluded if they are identified as ‘fatally flawed’ in the first instance, and included studies are assessed based on their quality and relevance.^40^ To develop this quality assessment tool, two researchers (CCA and KML) outlined criteria based on Dixon-Woods’ considerations of quality and relevance^40^ (see Supplementary file 3 for full quality assessment tool). Both researchers conducted quality assessment on an initial set of ten papers. Ratings were discussed, allowing discrepancies to be resolved through discussion and criteria to be iteratively refined. This process was repeated four times, until reasonable inter-rater reliability was reached. In total, 40 papers (36% of included papers) were assessed by both researchers, with 98% concordance reached at the final round. These papers were then revisited by both researchers to ensure that rating was consistent. The remaining papers were assessed by either CCA or KML.

 We used this approach to assess the diverse body of literature included in this review, including both qualitative and quantitative methods, many different study designs, as well as studies authored by researchers working in different disciplines with different approaches to reporting. In order to be inclusive of this range of literature, we only excluded papers as being ‘fatally flawed’ if their relevance was low, meaning that they did not meet the inclusion criteria.

 We analysed the charted data, presented a descriptive summary of the included studies in table form and identified intersections between STCS methods and domains of the policy process. We also conducted a thematic analysis of a subset of the included articles in order to identify what needs these methods have met and the resources they require, and what challenges were encountered in applying the methods. A random sample of the included articles was coded until new themes ceased to be identified. A purposive sample of additional articles was then coded, focusing on articles rated as having a ‘high’ relevance, as well as articles identified through the data charting process as having characteristics that were uncommon within the sample. Thematic analysis was conducted using the approach described by Braun and Clarke where the focus is guided by the researcher’s analytic interests,^41^ with four overarching themes chosen as an a priori coding framework: (1) value added or rationale for using STCS; (2) limitations of STCS methods; (3) challenges or barriers to using an STCS approach; and (4) characteristics that enabled or facilitated the use of an STCS approach. These four themes were chosen in light of our research questions and previous familiarisation with the included articles during the process of article selection, data extraction and quality assessment. Sub-themes were subsequently identified through close reading and coding of the included articles. Thematic analysis was coded by one researcher (CCA) using the qualitative data analysis software Dedoose^42^ and discussed with co-authors (KML and TLP).

## Results

**Figure 1:**
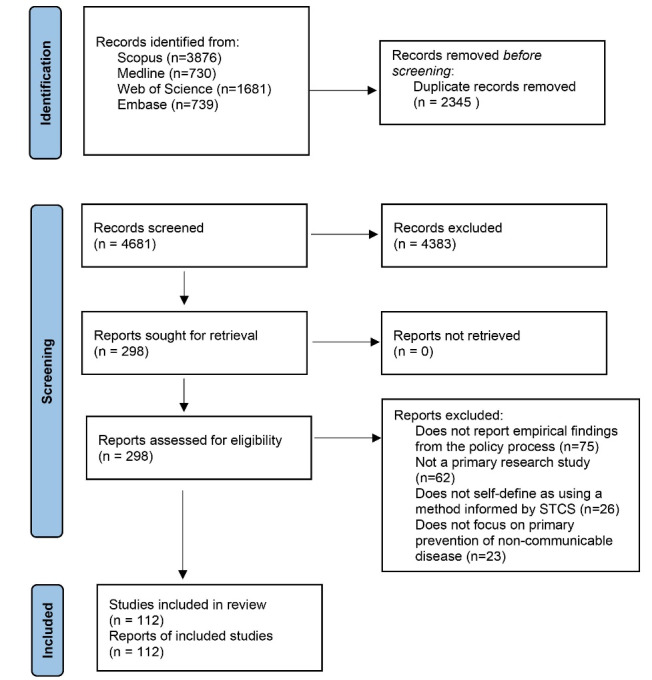
PRISMA 2020 Flow Diagram. Abbreviation: PRISMA, Preferred Reporting Items for Systematic Reviews and Meta-Analyses.

 After removing duplicates, our searches identified a total of 4681 records. After screening titles and abstracts, we considered 298 records for full-text review. In total, we included 112 papers in this review (Figure 1).^30^ Papers which concern potential risk factors for NCDs but focus on non-NCD outcomes (such as alcohol consumption as a risk factor for road traffic accidents or inter-personal violence) were excluded.

###  Use of STCS Methods in NCD Prevention Policy

**Figure 2:**
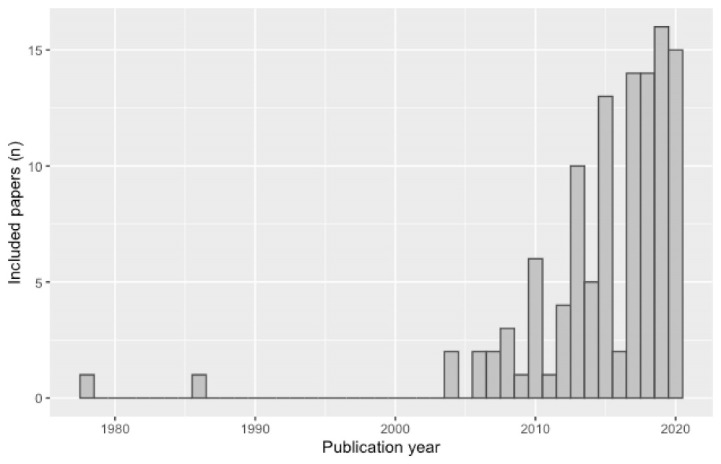
Papers Included in Review by Publication Year.

 The characteristics of the included studies are presented in Table 2. Studies focused on a number of different risk factors or health outcomes within NCD prevention, with the most common being tobacco (n = 18), obesity (n = 17) and physical activity (n = 12). Most studies were conducted in high-income countries, with a substantial number from the United States (n = 39), Australia (n = 20), Canada (n = 11), and New Zealand (n = 8). In addition, most studies had been published relatively recently, with 54% (n = 61) published after 2015 and 84% (n = 100) published after 2010 (Figure 2).

**Table 2:** Descriptive Characteristics of Included Studies (n = 112)

**Policy Process**	**Topic**	**First Author**	**Year**	**Policy Level**	**Country**	**Method**
Problem identification	Chronic disease	Willis^43^	2015	National	Canada	Concept mapping
	Chronic disease	Wutzke^14^	2017	National	Australia	Concept mapping
	Cardiovascular disease	Stankov^44^	2017	Local	Australia	Concept mapping
	Diabetes	Crespo^45^	2020	Local	Chile	Other (Spatial microsimulation and self-organising map)
	Diabetes	Giles^46^	2007	Local	Canada	Concept mapping
	Diet	Auchincloss^47^	2011	Multiple	USA	Agent-based modelling
	Diet	Gerritsen^48^	2019	National	New Zealand	Concept mapping
	Diet	Guariguata^49^	2020	National	Caribbean	Group model building
	Diet	Mazzocchi^50^	2020	Local	Italy	Systems lens
	Health promotion	Baugh^51^	2018	Local	Australia	Causal loop diagram
	NCD	Witter^52^	2020	Local	Sierra Leone	Group model building
	Nutrition	Roblin^53^	2018	Local	Canada	Systems lens
	Obesity in children	Nelson^54^	2015	Local	USA	Group model building
	Physical activity	Nau^55^	2019	National	Australia	Systems lens
	Public health	Malhi^56^	2009	Multiple	USA	Systems lens
	Sedentary behaviour	Buck^57^	2019	Regional	Europe	Network analysis
	Tobacco	Chao^58^	2015	National	Japan	Agent-based modelling
	Tobacco	Stillman^59^	2008	Regional	Southeast Asia	Concept mapping
	Urban health	Tan^60^	2019	Multiple	Malaysia	Causal loop diagram
	Urban health	Langellier^61^	2019	Local	Latin America	System dynamics model
Policy analysis	Active transport	Yang^62^	2013	Local	USA	Agent-based modelling
	Alcohol	Atkinson^63^	2018	Local	Australia	Agent-based modelling
	Alcohol	Castillo-Carniglia^64^	2018	Local	USA	Agent-based modelling
	Alcohol	Holder^65^	1987	Local	USA	System dynamics model
	Chronic kidney disease	Kang^66^	2018	Local	USA	System dynamics model
	Cardiovascular disease	Hirsch^67^	2010	National	USA	System dynamics model
	Cardiovascular disease	Li^68^	2015	Local	USA	Agent-based modelling
	Cardiovascular disease	Loyo^69^	2013	Local	USA	System dynamics model
Policy analysis	Cardiovascular disease	Yarnoff^70^	2019	Local	USA	System dynamics model
	Diabetes	Freebairn^71^	2020	National	Australia	Other (Hybrid simulation model)
	Diabetes	Freebairn^72^	2019	National	Australia	System dynamics model
	Diabetes	Li^73^	2017	Local	USA	Agent-based modelling
	Diet	Li^74^	2018	Local	USA	Agent-based modelling
	Diet	Widener^75^	2013	Local	USA	Agent-based modelling
	Drug (Pharmaceutical) policy	Abdollahiasl^76^	2014	National	Iran	System dynamics model
	NCD	Honeycutt^77^	2015	Local	USA	Other (ie, Prevention impacts simulation model)
	NCD	Signal^78^	2012	National	New Zealand	Systems lens
	Nutrition	Zhang^79^	2014	Local	USA	Agent-based modelling
	Obesity	Carrete^80^	2017	National	Mexico	System dynamics model
	Obesity	El-Sayed^81^	2012	National	England	Network analysis
	Obesity	Johnston^82^	2014	National	USA and Canada	Systems lens
	Obesity	Liu^83^	2016	National	USA	System dynamics model
	Obesity	Orr^84^	2015	National	USA	Agent-based modelling
	Obesity	Powell^85^	2017	Local	USA	System dynamics model
	Obesity	Roberts^86^	2019	National	Australia	System dynamics model
	Physical activity	Bellew^87^	2020	National	Australia	Concept mapping
	Physical activity	Browne^88^	2019	Local	Australia	Other (Spatial network analysis)
	Physical activity	Brennan^89^	2012	Local	USA	Concept mapping
	Physical activity	Yang^90^	2015	National	USA	Agent-based modelling
	Tobacco	Ahmad^91^	2007	National	USA	System dynamics model
	Tobacco	Cavana^92^	2008	National	New Zealand	System dynamics model
	Tobacco	Cavana^93^	2006	National	New Zealand	Group model building
	Tobacco	Hammond^94^	2020	Local	USA	Agent-based modelling
	Tobacco	Roberts^95^	1982	National	USA	System dynamics model
	Tobacco	Tengs^96^	2004	National	USA	System dynamics model
	Tobacco	Tengs^97^	2005	National	USA	System dynamics model
	Tobacco	Tobias^98^	2010	National	New Zealand	System dynamics model
Policy development	Aboriginal health	Browne^88^	2017	Local	Australia	Network analysis
	Active transport	Zwald^99^	2019	Local	USA	Network analysis
	Alcohol, obesity	Peters^100^	2017	Local	Netherlands	Network analysis
	Alcohol, obesity, diabetes	Freebairn^101^	2017	Local	Australia	System dynamics model
	Chronic disease	McGetrick^102^	2019	Local	Canada	Network analysis
	Cardiovascular disease	Garney^103^	2020	National	USA	Network analysis
	Diabetes	Beaton^104^	2019	Local	New Zealand	Systems lens
	Diet	Baker^105^	2019	Global	Global	Group model building
	Diet	Cullerton^106^	2017	National	Australia	Network analysis
	Environmental determinants of health	Peters^107^	2017	Local	Netherlands	Other (fuzzy set qualitative comparative analysis)
	Health equity	Heo^108^	2018	Local	South Korea	Network analysis
	Health equity	Scheele^109^	2018	Local	Scandinavia	Network analysis
	Obesity	Clarke^110^	2018	Local	Australia	Causal loop diagram
	Obesity	Clarke^111^	2020	Local	Australia	Causal loop diagram
	Obesity	Pérez-Escamilla^112^	2017	National	Latin America	Systems lens
	Obesity	Waqa^113^	2017	National	Fiji	Group model building
	Obesity	Cullerton^106^	2017	National	Australia	Network analysis
	Obesity	Sturgiss^114^	2019	Local	Canada	Concept mapping
	Obesity and diabetes	de Bruin^115^	2018	National	New Zealand	Network analysis
	Physical activity	Loitz^116^	2017	Local	Canada	Network analysis
	Physical activity	Barnes^117^	2010	Local	Canada	Network analysis
	Physical activity	Bergeron^118^	2014	Local	Canada	Concept mapping
	Physical activity	Buchthal^119^	2013	Local	USA	Network analysis
	Physical activity	Racine^120^	2020	Local	France	Concept mapping
	Physical activity	Spitters^121^	2017	Multiple	EU	Systems lens
	Population health	Leppin^122^	2018	Local	USA	Network analysis
	Public health	Fisher^123^	2016	Local	Australia	Systems lens
	Public health	Hoeijmakers^124^	2007	Local	Netherlands	Network analysis
	Aboriginal health	Leider^125^	2015	Local	USA	Network analysis
	Public health	Merrill^126^	2010	Local	USA	Network analysis
	Public health	Oliver^127^	2012	Local	UK	Network analysis
	Public health	Oliver^128^	2013	Local	UK	Other (mixed methods network analysis and qualitative interviews)
	Public health	Pineo^129^	2020	Local	Australia and USA	Causal loop diagram
	Public health	Mareeuw^130^	2015	Multiple	Netherlands	Systems lens
	Public health	Harris^131^	2013	Local	USA	Network analysis
	Tobacco	Harris^132^	2008	Local	USA	Network analysis
	Tobacco	Luke^133^	2013	Local	USA	Network analysis
	Tobacco	Moreland-Russell^134^	2015	Local	USA	Network analysis
	Tobacco	Weishaar^135^	2015	Regional	EU	Network analysis
	Tobacco	Weishaar^136^	2015	Regional	EU	Network analysis
Implementation	Active transport	Macmillan^137^	2020	Local	New Zealand	System dynamics model
	Health in all policies	Kokkinen^138^	2019	Multiple	Multiple	Systems lens
	Health in all policies	Shankardass^139^	2018	National	Finland	Systems lens
	Health promotion	Cambon^140^	2013	Multiple	Canada, France	Concept mapping
	Nutrition	Macdiarmid^141^	2011	National	UK	Concept mapping
	Obesity	Conte^142^	2020	Local	Australia	Other (Rich picture)
	Obesity	Roussy^143^	2019	Local	Australia	Systems lens
	Obesity	Beets^144^	2013	Multiple	USA	Systems lens
	Public health	Knai^145^	2018	National	UK	System dynamics model
	Public health	Kuunders^146^	2018	Local	Netherlands	Concept mapping
	Public health	Pagliccia^147^	2010	Local	Cuba	Network analysis
	Public health	van Roode^148^	2020	Local	Canada	Systems lens
	Tobacco	Terpstra^149^	2013	Regional	North America	Systems lens
	Tobacco	Valente^150^	2019	Global	Global	Network analysis
	Tobacco	Wen^151^	2020	Local	China	Network analysis

Abbreviation: NCD, Non-communicable disease.

Table 3 summarises the key STCS methods identified in the review.

**Table 3:** Key Methods Identified in the Review

**Method**	**Definition**	**Typical Applications in NCD Prevention Policy**	**Example Papers**
Systems lens	Qualitative or case study research informed by a systems perspective in terms of participant recruitment, data collection and/or data analysis	Developing an understanding of an existing policy landscape; identifying relevant stakeholders around a policy issue	^55,82^
System dynamics modelling	Method modelling stocks and flows within a system, where model structure can be informed by stakeholder input, quantitative data, and/or published theories and findings	Modelling policy scenarios to predict the impacts of different policy options on outcomes of interest	^92^
Network analysis	Method involving mapping connections between different actors (eg, individuals or organisations) with a system	Identifying relevant actors and the structure of their connections; understanding how social and organisational ties support or undermine policy progress	^152^
Group model building	Form of system dynamics modelling developed through participatory workshops	Modelling policy scenarios to predict the impacts of different policy options on outcomes of interest; consensus-building and stakeholder engagement	^93^
Concept mapping	Method involving the visual representation of different concepts and their relationships	Mapping existing systems of elements and actors to understand problems and current states	^59^
Causal loop diagram	Method mapping causal relationships between different elements within a system with a focus on characterising feedback loops	Modelling existing systems to understand problems and develop hypotheses around the potential impacts of policy options, particularly points at which impacts may be amplified or diminished over time	^129^
Agent-based modelling	Method modelling how individual agents react independently to hypothetical changes	Modelling policy scenarios to predict the impacts of different policy options on outcomes of interest in contexts where the behaviour of individual agents is key to outcomes	^75^

Abbreviation: NCD, Non-communicable disease.

**Figure 3:**
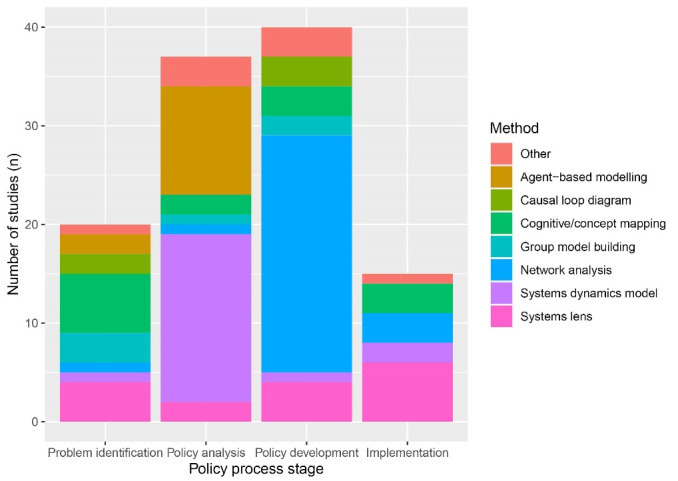
STCS Methods Used Across the Policy Process, Weighted by Number of Papers. Abbreviation: STCS, Systems thinking and complexity science.

 We identified recurring intersections between methods and policy process domains (Figure 3). System dynamics modelling and agent-based modelling, both methods typically used for quantitative modelling, were used more frequently for policy analysis. Network analysis, mainly focusing on relationships between individuals and organisations, was predominantly used for policy development. Concept mapping and qualitative research with a ‘systems lens’ were used consistently across the policy process. Meanwhile, group model building was used consistently for all domains of the policy process except implementation, while causal loop diagrams were used for problem identification and policy development.

 Studies focused on policy-making at different levels. Most studies were conducted in a local government context (n = 62) or at the national level (n = 35). A smaller number examined supra-national policy processes (n = 7). These studies included analyses of the role of international networks in European smoke-free policy^135,136^ and ‘policy diffusion’ across countries in the context of country-level implementation of the global Framework Convention on Tobacco Control.^150^ The remaining studies considered the policy process at multiple levels (n = 8), for example developing models,^47^ tools^140^ or frameworks^144^ that could be applied to inform or support policy-making in different contexts.

 Most of the included studies relied on stakeholder input (n = 87), including survey questionnaires, semi-structured interviews and participatory workshops. These stakeholders included policy-makers from several sectors, including health, transport, planning and education, as well as non-policy stakeholders such as community members, clinical practitioners, industry representatives and academics. The remaining studies used existing data sets or published literature in their analyses.

###  Quality Assessment

 The quality of included studies was high overall (see Supplementary file 4). Most studies were rated as high in terms of clearly stating their aims (n = 97), clearly identifying and justifying their research design (n = 102), and presenting their results clearly and comprehensively (n = 109). High ratings were slightly less frequent in terms of the reporting of the research process (n = 89) and analysis (n = 91). In terms of relevance, the studies were mixed, with just under half of studies having a high relevance (n = 50), indicating that the research was embedded in the policy process (commissioned, co-designed or conducted in partnership with government, or involving participatory workshops with stakeholders, see Supplementary file 3). The remaining papers (n = 62) were rated medium for relevance, meaning the studies were focused on understanding the policy process from an outside perspective.

###  Added Value of Using STCS Methods

 Authors cited a number of advantages to applying STCS methods to the policy process. Some advantages were mentioned across studies using different methods in different contexts. The participatory process inherent in some STCS methods was seen as a key feature, providing opportunities for building consensus among stakeholders from different sectors^59,146^ incorporating multiple perspectives when making decisions or trying to understand a problem; ^55,87,104,118^; and building capacity in terms of systems understanding among the policy or community stakeholders who participate in the research, for example through methods such as community-based system dynamics modelling.^61^ Other frequently noted advantages of STCS methods included facilitating evidence-informed decision-making;^45,92,152^ identifying ‘leverage points,’ or points within a system where interventions might be expected to be most impactful;^106,144,153^ integrating different forms of evidence;^57,63,101,145^ and tackling ‘wicked’ problems, where little progress had been made using other methods despite concerted efforts.^87,106,111,154^

 Other advantages were recognised as being specific to individual STCS methods. For example, the benefits of concept mapping included transparency of process and interpretability of results, making the method useful for engaging stakeholders with different areas of expertise.^59,146^ In turn, system dynamics modelling was seen as useful for modelling impacts on multiple outcomes^91,92^; surfacing mental models of systems^72^; and providing ‘dynamic decision support’ for policy decisions, offering a ‘what if’ tool to simulate different policies and explore their costs and benefits.^101^

 Finally, some advantages were frequently cited in studies focusing on specific domains of the policy process. In problem identification, involving clarifying and framing an issue in terms of its effect on population health, the suitability of STCS methods to modelling complex systems was noted.^45,47,57,58^ Systems that drive NCD-related practices such as smoking, physical activity and diet were understood as being complex and sometimes adaptive, being characterised by properties such as feedback loops and emergent behaviours. STCS methods are appropriate for modelling these types of systems and understanding the different drivers and outcomes of these practices.

 In policy analysis, which involves identifying and evaluating different policy options, STCS methods were useful in terms of enabling comparison of these different options through different types of modelling^81,92,155,156^; identifying potential unintended consequences^76,80,155^; and modelling impacts over different time scales.^63,80,81,93^ In addition, modelling different policy options was seen as a resource-efficient way to obtain information about their potential impacts, compared to implementing and evaluating policies.^63,81,155^

 In policy development, involving identifying a strategy for getting policies adopted, many authors used network analyses to understand how individuals and organisations relate to each other, influence each other, collaborate and share knowledge. In this context, STCS methods were useful in addressing multi-sectoral problems,^104,118,119,153^ and identifying disconnects between sectors or boundary-spanning organisations and individuals with the potential to connect disparate stakeholders.^88,119^

 Finally, in policy implementation, and particularly in evaluating policies that had been implemented, the potential of STCS methods to facilitate the incorporation of multiple perspectives was noted.^140,142^ This provided a more comprehensive understanding of how a policy operated within a complex system.

###  Limitations of STCS Methods

 Discussion of the limitations of STCS methods emphasised that, while these methods aim to incorporate more complexity than conventional methods, they still represent simplified versions of reality, and this must be acknowledged in interpreting findings.^64,75^ For example, the authors of a modelling study acknowledged model assumptions around potential policies having the same level of impact as reported in the literature.^85^ This ignored possible variation in impact caused by inadequate funding to implement policies and different approaches to monitoring and enforcement.^85^

 Authors also commented on the limitations of participatory methods: where different stakeholders were invited to jointly participate in workshops, their presence could have a silencing effect on other participants, for example senior and junior staff of an organisation.^148^ While some authors solved this issue by keeping these stakeholder groups separate,^130^ this may result in some of the advantages of a participatory method such as consensus- and capacity-building being lost.

###  Challenges or Barriers to Using an STCS Approach

 Authors also encountered challenges while applying STCS-informed methods to NCD prevention policy. Along with challenges encountered in applying research methods of any kind, such as a lack of appropriate data^47,63,76,81,93^ and high requirements in terms of technical skill,^81,93,142^ studies also noted particular challenges in applying STCS methods, mainly relating to engaging stakeholders in the research process.

 First, although the participatory processes involved in some STCS methods were noted as a key source of added value, implementing these processes was also challenging. Challenges included the resource-intensiveness of participatory processes, with both time required of participants and travel costs being high^51,89,118,142^; maintaining communication and engagement with participants throughout a sometimes lengthy research process^87,101^; and different expectations and priorities among participants.^101,142^

 Second, researchers also noted a lack of trust in models as a source of evidence, with modelling evidence not always seen as strong enough to act on.^72,101^ To some extent, participatory modelling was seen as a way to add credibility to models, as stakeholders were embedded in the modelling process and developed a better understanding of how models incorporate and generate evidence.

 Third, authors also noted that in some cases both processes and results were too complex to engage some stakeholder groups, and stakeholders struggled to interpret them.^47,76,81,140^ Some authors noted a tension around model complexity, wanting models to be both complex enough to adequately represent the system in question, and simple enough to lend themselves more readily to interpretation.^47,76,81^

###  Characteristics That Enabled or Facilitated the Use of an STCS Approach

 While authors noted a number of challenges which were encountered in applying STCS methods to the policy process, they also provided many examples of how to make these methods work in practice.

 First, many studies adapted methods to make them less resource-intensive, particularly in terms of demanding less of their participants. For example, a number of studies used online platforms to engage participants and collect data, allowing geographically dispersed participants to participate without the need to travel.^89^ Some studies also enabled participants to contribute asynchronously, for example through qualitative interviews, which were then integrated with other perspectives by the research team.^48^

 Second, authors noted the importance of presenting processes and results in an accessible way, allowing different groups of stakeholders to engage with and learn from them. For example, one study reporting system dynamics modelling of diabetes in pregnancy described the use of individual ‘life stories’ as a means of communicating model structure and results: hypothetical patient journeys were described in light of the model, with patients being born with particular risk profiles, ageing over the course of their lives, gaining and losing weight, undergoing clinical procedures or changes in health-related practices, and so on.^72^ This helped make the modelling process and findings relevant to different stakeholder groups. Other approaches included the use of ‘rich pictures’ to communicate complex findings,^142^ or interactive user interfaces allowing stakeholders to model different policy interventions for themselves.^69,73,77,93,101^ Authors also cited progress in modelling software as an enabling factor, making it more user-friendly for different stakeholders.^63,71^

 Third, authors emphasised the importance of fostering stakeholder ownership of the research process.^72,101^ This involved ensuring that stakeholders identified, or at least acknowledged, the problem under consideration as important, as well as in some cases being an issue that ‘conventional’ approaches had failed to address. Another key aspect was recruiting participants who had credibility, which facilitated buy-in from stakeholders who were not involved in the research process, as participants acted as ‘ambassadors’ for the process. Stakeholder ownership could also be encouraged through the facilitation process, for example by highlighting participants’ contributions to different parts of the model, allowing them to see how their input was incorporated.

 A number of other characteristics that enabled these methods to be put to use, including ensuring expert facilitation of any workshops^72,101^; transparency and acknowledgement around the limitations of STCS methods,^72,101^ with authors acknowledging that, even though STCS methods seek to incorporate a greater degree of complexity, complexity is still lost in the modelling process^75^; and insider access to relevant stakeholder groups.^111^

## Discussion

###  Implications for Research and Practice

 This study presents a comprehensive review of how methods informed by STCS have been applied in the policy process in NCD prevention. The findings can inform researchers and policy-makers around which methods are most frequently applied to both facilitate and understand different domains of the policy process (Figure 3), as well as typical applications of these methods in NCD prevention policy (Table 3). This overview of current practice may be helpful when selecting the most appropriate STCS method to address a particular research objective. The study also sheds light on the practical dimensions of applying these methods, including considerations of the added value of STCS methods in the policy process; the limitations of these methods; the challenges that have been encountered in applying these methods; and the circumstances that have facilitated their application. A particular finding for practice is the importance of stakeholder engagement when using STCS methods in policy-making. As several of the methods identified in this review involve some form of system modelling, particularly in comparing scenarios at the policy analysis stage, authors highlight stakeholders’ reluctance to use models and their evidence to support policy decisions. However, stakeholder engagement in model conceptualisation and development emerges as key to building trust in models.

###  Comparison With Existing Literature

 To our knowledge, this is the first systematic scoping review identifying and analysing the range of STCS-informed methods applied in the policy process for NCD prevention. In line with previous work, we found that STCS-informed methods were valued as a way of building consensus between different stakeholders in the policy context,^16^ and changing policy-makers’ perspectives on their work.^24^ Previous work also identified a lack of trust in models as a form of evidence as a barrier to use.^16^ In turn, our findings present approaches to building trust, providing examples of how processes and results can be communicated in user-friendly ways and emphasising the importance of stakeholder ownership. This latter finding echoes Rouwette’s review of group model building case studies in a wide range of settings, which emphasises ownership of the model and trust in the modeller as an important outcome of the group model building process.^9^

 In contrast to existing reviews around system dynamics modelling in health policy^16^ and systems thinking in public health more broadly,^2,18^ which noted a paucity of practical applications and the need for additional applied work, we identified a substantial number of examples, reflecting our broader scope in terms of methods as well as the increasing emphasis on these methods in the literature in recent years (see Figure 2). The numerous applied examples identified in this review supported us in developing insights around the added value and limitations of, as well as barriers and facilitators to, using STCS methods in NCD prevention policy. However, in common with previous work on the topic,^16,18^ we identified a lack of evaluations of effectiveness of policy decisions made as a result of STCS-informed methods. While previous reviews highlighted that system dynamics modelling studies often claimed a greater validity due to being based on systems principles, without providing evidence of this validity in the form of, for example, greater predictive power than conventional models,^16,18^ we found this pattern repeated across the range of STCS methods included in this review. While researchers and funders may hesitate to use both conventional and STCS methods to answer the same question, particularly due to the often larger resource requirements of STCS studies, such comparative work would help clarify where STCS methods are useful and where they are not. This remains an important gap in the evidence base.

###  Strengths and Limitations of the Review

 This review could only identify examples of methods which have previously been applied in the policy domain, and where this application was documented. We hope this will increase the value of our findings for practitioners, but as a result, methods that have not been applied, or only applied in other fields, could not be identified in this review.

 Further, in an attempt to include the wide range of STCS methods in use, we searched for and included studies using specific methods previously identified as being informed by STCS (eg, concept mapping, system dynamics modelling, network analysis),^16,17,157^ as well as studies whose authors characterised their work as being informed by STCS. As a result, studies that neither employed methods identified by existing literature as being informed by STCS, nor ‘self-defined’ as using methods informed by STCS, were not included. Anzola and colleagues highlight the existence of ‘analogical’ uses of terms relating to complexity, where central characteristics of STCS are employed or implicitly referred to without being explicitly linked to the relevant theory and methods.^158^ In the absence of shared terminology, such usage may be difficult to systematically identify in the literature. However, given that the aim of this review was to identify the applications of specific methods, we aimed to be inclusive of relevant theories and methods. In addition, the inclusion of additional terms relating to NCD prevention, such as ‘population health,’ may have yielded additional relevant examples.

 Finally, this review focused on peer-reviewed literature in order to identify ‘best practice,’ methodologically robust descriptions of the specific and distinct methods that are in use. As a result, applications of methods informed by STCS which are documented in the grey literature were not identified, potentially excluding methods used in non-academic settings, such as government institutions, non-government organisations, and public health practice.

 In conducting this review, we developed a quality assessment tool informed by an existing approach to assessing and synthesising complex, multi-disciplinary bodies of literature. Both authors who conducted quality assessment have backgrounds in public health and epidemiology, and may have been biased towards their own disciplinary standards of reporting. As a result, some studies, while presenting interesting ideas and arguments, ranked low in terms of quality. In order not to exclude papers due to disciplinary differences, all papers were included other than those that ranked low in terms of relevance to our research questions, meaning they did not meet our inclusion criteria. The ‘relevance’ criterion was used to inform our analysis of included papers, as described in the methods. This is an initial attempt at quality assessment in this body of literature, but future research may refine this process further by considering disciplinary differences in reporting.

###  Unanswered Questions and Future Research

 This study identifies some of the ways in which, from the perspective of study authors, methods informed by STCS add value throughout the policy process. However, more formal analysis of the added value of these methods could be an important next step in determining their usefulness. While the effectiveness of some systems methods has been reviewed, review authors cite a lack of uniformity in approaches to determining ‘effectiveness.’^9^ Contributing to our understanding of effectiveness would require some consideration of what stakeholders expect of these methods. For example, the focus might be on outcomes, considering whether policies incorporating STCS into their design and implementation might be more effective in achieving desired outcomes. On the other hand, the impact on the policy process itself might be deemed important, with participatory STCS methods perhaps being more inclusive of multiple perspectives.

 Our findings also highlight contexts in which methods informed by STCS have seen more limited application in policy-making for NCD prevention, particularly in low- and middle-income countries and in policy-making at the supra-national level. Future research could seek to apply these methods in these contexts and document the process, identifying whether sources of value added, challenges encountered or facilitating characteristics are similar or different.

## Conclusion

 This systematic scoping review aimed to identify existing applications of STCS methods to the policy process in NCD prevention. A substantial number of studies were identified, exhibiting diversity in terms of method, level of government, area of NCD prevention, and policy process domain. The findings of this review can inform researchers and policy-makers around which methods are most frequently applied to both facilitate and understand different domains of the policy process, as well as providing practical insights around their application. Future research in this area could focus on applying these methods in policy-making in low- and middle-income countries and at the supra-national level, as well as generating additional evidence around the added value of using these methods in NCD prevention policy.

## Ethical issues

 This study involved a synthesis of existing peer-reviewed literature and no primary data were collected. Ethical approval was therefore not required.

## Competing interests

 Authors declare that they have no competing interests.

## Authors’ contributions

 Conception and design: TLP, CCA, EM, and ME. Acquisition of data: CCA, TLP, and EM. Analysis and interpretation of data: CCA, KML, and JC. Drafting of the manuscript: CCA. Critical revision of the manuscript for important intellectual content: KML, EM, ME, RM, HR, AH, KW, and TLP. Obtaining funding: TLP.

## Funding

 CCA, TLP, and JC acknowledge internal research support from York University, Toronto, Canada. CCA and TLP acknowledge funding from the WHO European Office for Prevention and Control of Noncommunicable Diseases. EM and ME were supported by the National Institute for Health Research (NIHR) School for Public Health Research (SPHR), Grant Reference Number PD-SPH-2015.

## Supplementary files


Supplementary file 1. Example Search Strategy.

Supplementary file 2. Data Charting Form.

Supplementary file 3. Quality Assessment Tool.

Supplementary file 4. Quality Assessment Results.
