# Supplementary material for: Systems Thinking and Complexity Science Methods and the Policy Process in Non-communicable Disease Prevention: A Systematic Scoping Review
Source: Int J Health Policy Manag. 2023 Feb 26;12:6772. doi: 10.34172/ijhpm.2023.6772 (PMC10125079; doi:10.34172/ijhpm.2023.6772)
Supplement: Supplementary file 1 — Example Search Strategy. [file ijhpm-12-6772-s001.pdf]

Article title: Systems Thinking and Complexity Science Methods and the Policy Process in Non-communicable Disease Prevention: A Systematic Scoping Review

Journal name: International Journal of Health Policy and Management (IJHPM)

Authors' information: Chloe Clifford Astbury[1], Kirsten M. Lee[1], Elizabeth McGill[2], Janielle Clarke[1], Matt Egan[3], Afton Halloran[4,5], Regina Malykh[4], Holly Rippin4, Kremlin Wickramasinghe[4], Tarra L. Penney[1]*

[1]Global Food System & Policy Research, School of Global Health, York University, Toronto, ON, Canada.

[2]Department of Health Services Research and Policy, London School of Hygiene & Tropical Medicine, London, UK.

[3]Department of Public Health, Environments and Society, London School of Hygiene & Tropical Medicine, London, UK.

[4]World Health Organization European Office for the Prevention and Control of Noncommunicable Diseases, Moscow, Russian Federation.

[5]Department of Nutrition, Exercise and Sports, University of Copenhagen, Copenhagen, Denmark.

(*Corresponding author: tpenney@yorku.ca)

Supplementary file 1. Example Search Strategy

Search strategy developed for Scopus

TITLE-ABS-KEY (("system theory" OR "system thinking" OR "system science" OR "complex system" OR "system model" OR "system dynamics" OR "system approach" OR "system lens" OR "system perspective" OR complexity OR "complexity theory" OR "complexity science" OR "adaptive system" OR "soft system" OR "agent-based model" OR "group model building" OR "concept mapping" OR "system dynamic" OR "network analysis" OR "partial model testing" OR "system heuristics" OR "system heuristics" OR "causal loop diagram" OR "scenario technique" OR cynefin OR "solution focus" OR behavior-over-time OR "discrete event modelling") AND (policy OR law OR legal OR legislative OR regulation OR regulate OR regulatory OR tariff OR subsidy OR tax OR ban OR "voluntary agreement" OR incentive OR fiscal OR guidelines OR government) AND (evaluation OR implementation OR facilitation OR "policy development" OR policymaking OR "case study" OR "problem identification" OR "decision-making" OR strategy OR "policy enactment" OR "policy analysis" OR "stakeholder engagement") AND ("public health" OR "health promotion" OR "health inequality" OR "health inequity" OR "health behavior" OR "well being" OR wellbeing OR nutrition OR diet OR obesity OR "fast food" OR "junk food" OR sugar OR salt OR tobacco OR smoking OR cigarette OR alcohol OR "illegal drug*" OR "illicit drug" OR "recreational drug" OR "social determinant" OR "physical activity" OR exercise OR "non-communicable disease" OR "noncommunicable disease" OR "chronic disease" OR "sedentary behaviour" OR NCD) AND NOT (gene OR genetic OR transcript OR transcription OR cell OR nucleus OR mouse OR mice))
