# Supplementary material for: Systems Thinking and Complexity Science Methods and the Policy Process in Non-communicable Disease Prevention: A Systematic Scoping Review
Source: Int J Health Policy Manag. 2023 Feb 26;12:6772. doi: 10.34172/ijhpm.2023.6772 (PMC10125079; doi:10.34172/ijhpm.2023.6772)
Supplement: Supplementary file 2 — Data Charting Form. [file ijhpm-12-6772-s002.pdf]

Article title: Systems Thinking and Complexity Science Methods and the Policy Process in Non-communicable Disease Prevention: A Systematic Scoping Review

Journal name: International Journal of Health Policy and Management (IJHPM)

Authors' information: Chloe Clifford Astbury[1], Kirsten M. Lee[1], Elizabeth McGill[2], Janielle Clarke[1], Matt Egan[3], Afton Halloran[4,5], Regina Malykh[4], Holly Rippin4, Kremlin Wickramasinghe[4], Tarra L. Penney[1]*

[1]Global Food System & Policy Research, School of Global Health, York University, Toronto, ON, Canada.

[2]Department of Health Services Research and Policy, London School of Hygiene & Tropical Medicine, London, UK.

[3]Department of Public Health, Environments and Society, London School of Hygiene & Tropical Medicine, London, UK.

[4]World Health Organization European Office for the Prevention and Control of Noncommunicable Diseases, Moscow, Russian Federation.

[5]Department of Nutrition, Exercise and Sports, University of Copenhagen, Copenhagen, Denmark.

(*Corresponding author: tpenney@yorku.ca)

Supplementary file 2. Data Charting Form

Table A1: Data charting form

**Record**	Title
	First author
	Year
**Application**	Policy process (problem identification, policy analysis, strategy and policy development, policy enactment, policy implementation) (149)
	Evaluation (yes/no)
	Stakeholder engagement or education (yes/no)
	Area of NCD prevention (health outcome or risk factor)
	Policy level (local, national, regional, global)
	Stakeholders involved, if any (government, academic, professional, industry, community)
	Project (state if publication was part of a larger project)
	Country
**Method**	Name
	Tool used (if any: software, kit)
	Aim/research question (if stated)
