# Supplementary material for: Systems Thinking and Complexity Science Methods and the Policy Process in Non-communicable Disease Prevention: A Systematic Scoping Review
Source: Int J Health Policy Manag. 2023 Feb 26;12:6772. doi: 10.34172/ijhpm.2023.6772 (PMC10125079; doi:10.34172/ijhpm.2023.6772)
Supplement: Supplementary file 3 — Quality Assessment Tool. [file ijhpm-12-6772-s003.pdf]

Article title: Systems Thinking and Complexity Science Methods and the Policy Process in Non-communicable Disease Prevention: A Systematic Scoping Review

Journal name: International Journal of Health Policy and Management (IJHPM)

Authors' information: Chloe Clifford Astbury[1], Kirsten M. Lee[1], Elizabeth McGill[2], Janielle Clarke[1], Matt Egan[3], Afton Halloran[4,5], Regina Malykh[4], Holly Rippin4, Kremlin Wickramasinghe[4], Tarra L. Penney[1]*

[1]Global Food System & Policy Research, School of Global Health, York University, Toronto, ON, Canada.

[2]Department of Health Services Research and Policy, London School of Hygiene & Tropical Medicine, London, UK.

[3]Department of Public Health, Environments and Society, London School of Hygiene & Tropical Medicine, London, UK.

[4]World Health Organization European Office for the Prevention and Control of Noncommunicable Diseases, Moscow, Russian Federation.

[5]Department of Nutrition, Exercise and Sports, University of Copenhagen, Copenhagen, Denmark.

(*Corresponding author: tpenney@yorku.ca)

Supplementary file 3. Quality Assessment Tool

Only papers that had a 'low' relevance to the research question were deemed fatally flawed and excluded. All other combinations of ratings were included.

Table A2: Quality assessment criteria

Criterion	Rating	Definition
Quality		
Clarity of aims and objectives: are the aims clearly stated?	High	Aims are explicitly stated and specific
	Medium	Aims are not explicitly stated but can be inferred, or are stated but are vague (e.g. describe process rather than intention)
	Low	Aims are not stated and cannot be inferred

Appropriateness of research design: do the authors justify why the research design (e.g. network analysis, ABM, GMB) is appropriate to answer their question?	High	Clear, explicit justification for choosing a method
	Medium	Method choice was not explicitly justified but appears suitable to the research question
	Low	Method not justified by authors and does not appear to fit the research question
Clarity of research process: is participant recruitment and data collection (e.g. questionnaires, workshop scripts/schedules) clearly described?	High	Research process is explicitly stated, clear and specific. Decisions around research process (e.g. data collection, participant recruitment) are clearly described and justified
	Medium	Research process is described imprecisely or lacking in detail
	Low	Research process is described with little or no data or not at all. Specific source of data cannot be inferred from the manuscrupt
Clarity of analysis: are the data analysis procedures clearly described?	High	Analysis is explicitly stated, clear and specific. Decisions around analysis (i.e. method, statistical tests) are clearly described and justified
	Medium	Analysis is described imprecisely or lacking in detail
	Low	Analysis is described with little or no detail or not at all. Analysis approach cannot be inferred from the manuscript
Sufficiency of data to support interpretations and conclusions: are results presented clearly and comprehensively?	High	Results are clear and comprehensive (i.e. authors present/describe their data set and the results of their analyses)

	Low	Results are described with little or no detail or not at all
	Relevance	
Relevance to research question	High	Research is embedded in the policy process (e.g. commissioned, co-designed or conducted in partnership with government, or involving participatory workshops with stakeholders)
	Medium	Other applications of systems methods in the policy space
	Low	Papers do not meet inclusion criteria; exclude
