# Supplementary material for: Systems Thinking and Complexity Science Methods and the Policy Process in Non-communicable Disease Prevention: A Systematic Scoping Review
Source: Int J Health Policy Manag. 2023 Feb 26;12:6772. doi: 10.34172/ijhpm.2023.6772 (PMC10125079; doi:10.34172/ijhpm.2023.6772)
Supplement: Supplementary file 4 — Quality Assessment Results. [file ijhpm-12-6772-s004.pdf]

Article title: Systems Thinking and Complexity Science Methods and the Policy Process in Non-communicable Disease Prevention: A Systematic Scoping Review

Journal name: International Journal of Health Policy and Management (IJHPM)

Authors' information: Chloe Clifford Astbury[1], Kirsten M. Lee[1], Elizabeth McGill[2], Janielle Clarke[1], Matt Egan[3], Afton Halloran[4,5], Regina Malykh[4], Holly Rippin4, Kremlin Wickramasinghe[4], Tarra L. Penney[1]*

[1]Global Food System & Policy Research, School of Global Health, York University, Toronto, ON, Canada.

[2]Department of Health Services Research and Policy, London School of Hygiene & Tropical Medicine, London, UK.

[3]Department of Public Health, Environments and Society, London School of Hygiene & Tropical Medicine, London, UK.

[4]World Health Organization European Office for the Prevention and Control of Noncommunicable Diseases, Moscow, Russian Federation.

[5]Department of Nutrition, Exercise and Sports, University of Copenhagen, Copenhagen, Denmark.

(*Corresponding author: tpenney@yorku.ca)

Supplementary file 4. Quality Assessment Results

Table A3: Results of quality assessment for included studies (using criteria defined in Appendix 3)

**Authors**	**Year**	**Clarity of aims and objectives**	**Appropriateness of research design**	**Clarity of research process**	**Clarity of analysis**	**Sufficiency of data to support interpretations and conclusions**	**Relevance to research question**
Abdollahiasl et al.	2014	High	High	Low	Medium	High	Medium
Ahmad et al.	2007	High	High	High	High	High	Medium
Atkinson et al.	2017	High	High	High	High	High	High
Auchincloss et al.	2011	High	High	High	High	High	Medium
Baker et al.	2019	High	High	High	Medium	High	High
Barnes et al.	2010	High	High	High	Medium	High	Medium
Baugh Littlejohns et al.	2018	Medium	High	High	High	High	Medium
Beaton et al.	2019	High	High	Medium	High	High	Medium
Beets et al.	2013	Medium	Low	Low	Low	Low	Medium
Bellew et al.	2020	High	High	Medium	Low	Medium	High
Bergeron et al.	2014	High	High	High	High	High	High
Brennan et al.	2012	High	High	High	High	High	High
Brown et al.	2019	High	High	High	High	High	Medium
Browne et al.	2016	High	High	High	High	High	Medium
Buchthal et al.	2013	High	High	High	High	High	Medium
Buck et al.	2019	High	High	High	High	High	Medium
Cambon et al.	2013	High	High	Medium	High	High	Medium
Carrete et al.	2017	High	High	High	High	High	High
Castillo-Carniglia et al.	2018	High	High	High	High	High	Medium
Cavana and Tobias	2008	High	High	Low	Medium	High	Medium

Cavana et al.	2006	High	Medium	Medium	Medium	High	High
Chao et al.	2015	High	High	High	High	High	Medium
Clarke et al.	2018	Medium	Medium	High	High	High	Medium
Clarke et al.	2020	High	High	High	High	High	Medium
Conte et al.	2020	High	High	High	High	High	High
Crespo et al.	2020	High	High	High	High	High	Medium
Cullerton et al.	2017	High	High	High	High	High	High
Cullerton et al.	2016	High	High	High	High	High	High
de Bruin et al.	2018	High	High	High	High	High	Medium
El-Sayed et al.	2012	High	High	High	High	High	Medium
Fisher et al.	2014	High	High	High	High	High	High
Freebairn et al.	2017	High	High	High	High	High	High
Freebairn et al.	2020	High	High	High	High	High	High
Freebairn et al.	2019	High	High	High	High	High	High
Garney et al.	2020	High	High	High	High	High	High
Gerritsen et al.	2019	High	High	High	High	High	High
Giles et al.	2006	High	High	High	High	High	Medium
Guariguata et al.	2020	High	High	Medium	High	High	High
Hammond et al.	2020	High	High	High	High	High	Medium
Harris	2013	High	High	High	High	High	Medium
Harris et al.	2008	High	High	High	High	High	Medium
Heo et al.	2018	High	High	High	High	High	High
Hirsch et al.	2010	Medium	High	High	High	High	Medium
Hoeijmakers et al.	2007	High	Medium	Medium	Medium	High	High
Holder et al.	1986	High	High	High	High	High	Medium
Honeycutt et al.	2015	High	High	High	High	High	High
Johnston et al.	2014	High	High	High	High	High	Medium
Kang et al.	2018	High	High	High	High	High	Medium
Knai et al.	2018	High	High	High	High	High	Medium
Kokkinen et al.	2019	High	High	High	High	High	Medium
Kuunders et al.	2018	High	High	High	High	High	High
Langelier et al.	2019	High	High	High	High	High	High
Leider et al.	2015	High	High	High	High	High	Medium
Leppin et al.	2018	High	High	High	High	High	High
Li et al.	2017	High	High	High	High	High	Medium
Li et al.	2018	High	High	High	High	High	Medium
Li et al.	2015	High	High	High	High	High	Medium
Liu et al.	2015	High	High	High	High	High	Medium
Loitz et al.	2017	High	High	High	High	High	High
Loyo et al.	2013	Medium	High	Medium	Medium	High	High
Luke et al.	2013	High	High	High	High	High	High
Macdiarmid et al.	2010	High	High	High	High	High	High
Macmillan et al.	2020	High	High	High	High	High	High
Mahli et al.	2009	Medium	Medium	Medium	High	High	Medium
Mazzocchi et al.	2020	Low	Low	Medium	Low	High	High
McGetrick et al.	2019	Medium	High	High	High	High	Medium
Merrill et al.	2010	High	High	High	High	High	High

Moreland-Russell et al.	2015	High	High	High	High	High	Medium
Nau et al.	2019	High	High	High	Medium	High	High
Nelson et al.	2015	High	High	Medium	Low	Low	High
Oliver et al.	2012	Medium	High	High	High	High	Medium
Oliver et al.	2013	High	High	High	High	High	High
Orr et al.	2015	High	High	High	High	High	Medium
Pagliccia et al.	2010	Medium	High	High	Low	High	Medium
Pérez-Escamilla et al.	2017	High	Medium	Medium	Medium	High	High
Peters et al.	2017	High	High	Medium	High	High	Medium
Peters et al.	2017	High	High	High	High	High	High
Pineo et al.	2020	High	High	High	High	High	High
Powell et al.	2017	High	High	High	High	High	High
Racine et al.	2020	High	High	High	High	High	High
Roberts et al.	2018	High	High	High	High	High	High
Roberts et al.	1978	High	High	Medium	High	High	Medium
Roblin et al.	2018	High	Medium	Medium	Low	High	High
Roussy et al.	2019	High	High	High	High	High	Medium
Scheele et al.	2018	Medium	High	High	High	High	High
Shankardass et al.	2018	High	High	Low	Low	High	Medium
Signal et al.	2012	High	High	High	High	High	High
Spitters et al.	2017	High	High	High	High	High	High
Stankov et al.	2017	High	High	High	High	High	High
Stillman et al.	2008	High	High	High	High	High	High
Sturgiss et al.	2019	High	High	Medium	Medium	High	Medium
Tan et al.	2019	Medium	High	Medium	Medium	High	High
Tengs et al.	2004	Medium	Medium	High	High	High	Medium
Tengs et al.	2004	High	High	High	High	High	Medium
Terpstra et al.	2013	High	High	High	High	High	High
Tobias et al.	2010	High	High	High	High	High	Medium
Valente et al.	2019	High	High	High	High	High	Medium
van den Driessen Mareeuw et al.	2015	High	High	High	High	High	Medium
van Roode et al.	2020	High	High	Medium	High	High	Medium
Waqa et al.	2017	High	High	High	Low	High	High
Weishaar et al.	2015	High	High	Medium	Medium	High	Medium
Weishaar et al.	2015	High	High	High	High	High	Medium
Wen et al.	2020	Medium	High	High	High	High	Medium
Widener et al.	2013	High	High	High	High	High	Medium
Willis et al.	2015	Medium	Medium	Medium	Medium	High	High
Witter et al.	2020	High	High	High	High	HIgh	High
Wutzke et al.	2017	High	High	High	High	High	Medium
Yang et al.	2013	High	High	High	High	High	Medium
Yang et al.	2015	High	High	High	High	High	Medium
Yarnoff et al.	2019	High	High	High	High	High	Medium
Zhang et al.	2014	High	High	High	High	High	Medium
Zwald et al.	2019	High	High	High	High	High	Medium
